# Evaluating potential direct and carry-over weather effects on production performance in a divergently selected Merino flock

**DOI:** 10.1007/s00484-025-02946-z

**Published:** 2025-05-28

**Authors:** P. G. Theron, T. S. Brand, S. W. P. Cloete, J. H. C. van Zyl

**Affiliations:** 1 Department of Animal Sciences, Stellenbosch University, Private Bag X1, Matieland, 7602 South Africa; 2 Directorate: Animal Sciences, Department of Agriculture, Western Cape Government, Private Bag X1, Elsenburg, 7607 South Africa

**Keywords:** Climate change, Decision support system, Environmental effects, Regression models

## Abstract

Climate change and the associated changing weather patterns provide a global challenge to livestock producers. Due to the lack of information on the exact relationship between weather patterns and production output, livestock producers may struggle to adjust to these changing environmental conditions. This study therefore evaluated the feasibility of modelling the impact of weather conditions both within and across production seasons on production output in two divergently selected lines of Merino ewes. Production data collected from the high and low line of the Elsenburg Merino flock between 1993 and 2021 were related to weather data recorded by a weather station on the farm. The weather data included temperature, relative humidity and rainfall readings. Multiple linear regressions between uncorrelated weather variables and production parameters (conception, lambing, multiple offspring and survival percentages, average weaning weight and weight weaned per ewe) were derived to quantify the relationship between weather and production. Within production season, weather conditions during mating significantly affected lambing (R^2^ = 0.332) and multiple offspring percentages (R^2^ = 0.316) in the high line and lambing percentage (R^2^ = 0.205) in the low line. Lambing period weather affected average weaning weight in the high line (R^2^ = 0.230) and survival percentage in the low line (R^2^ = 0.193). Significant amounts of the variation (R^2^ = 0.180–0.306) in various production traits for both lines were also accounted for by the fitting of regression models of weather conditions in the preceding year to current production performance. This indicates the presence of weather-related carry-over effects. It therefore appears that using weather data to predict production output, both within and between production seasons, may be a viable management tool to aid producers in decision making. More work is required before these models are suitable for general uptake.

## Introduction

In South Africa, most land designated for agricultural use is considered non-arable (FAOSTAT 2020), leaving grazing-based livestock farming as the only viable agricultural enterprise that can be practiced in these areas (Cloete and Olivier 2010). This has naturally led to many extensive livestock producing enterprises, primarily sheep farms, arising particularly in the drier western part of the country (Cloete and Olivier 2010). Sheep are also commonly farmed in ley farming or mixed crop-livestock systems in cropping regions as they help to buffer farm income against fluctuations in crop production or prices (Cloete and Olivier 2010). Sheep in such systems are also maintained under extensive conditions for most of the year. The adaptability and suitability of sheep to all these farming systems has led to sheep farming being widely practiced across South Africa (DALRRD 2022). Historically speaking, sheep farming has also played an important role in South African agriculture with nomadic tribes keeping sheep flocks as early as 200–400 AD (Snyman 2014). European settlers introduced further breeds to the region, most notably the Merino (Bath 2023), which is today considered to be the most important wool-producing breed in the country (DALRRD 2022) and contributes significantly to both meat and wool income from the national flock (DALRRD 2022; Cape Wools 2023).

Extensive production systems, where most of these animals are farmed in, are extremely reliant upon annual precipitation levels to ensure sufficient fodder availability (Li et al. 2012) and are therefore highly susceptible to weather effects, particularly droughts (Rust and Rust 2013; Oduniyi et al. 2020). This applies both to animals kept on natural grazing and those on crop residues. Weather and climate may further affect production, health and welfare of farm animals negatively in various ways (Nardone et al. 2006). Prolonged increases in environmental temperatures can lead to animals experiencing heat stress, which could impair production (Rust and Rust 2013; Gowane et al. 2017; Joy et al. 2020). According to Bohmanova et al. (2007), heat stress is influenced by a number of environmental factors including temperature, relative humidity, precipitation, solar radiation and air movement. This clearly shows that weather patterns could have a significant impact on production output in extensive systems.

This is of concern to livestock producers because of the global trend that annual mean temperatures are increasing and look set to continue doing so (IPCC 2021). In South Africa this acceleration has been faster than the average (Ziervogel et al. 2014; Pereira 2017) and projections indicate that this trend will continue (Gizaw and Gan 2017; Jury 2013; Meissner et al. 2013; Niang et al. 2014; Pereira 2017; Scholtz et al. 2016). Furthermore, the increase in extreme warm indices coupled with a concurrent decrease in extreme cold indices in Southern Africa indicates a warming pattern (Pereira 2017). Since farming activities are reliant upon favourable climatic and environmental conditions (Porter et al. 2014), producers could face challenges in future if these projections are realized.

Producers firstly need information on the impact these projected changes would have on their production systems to overcome the expected challenges. Secondly, they require some way of proactively applying this information to decision making and management to improve the sustainability and resilience of their enterprises. Currently neither of these needs are being met because, although a large body of research on expected climate change exists (Meissner et al. 2013; Jury 2013; Niang et al. 2014; Ziervogel et al. 2014; Scholtz et al. 2016; Pereira 2017; Gizaw and Gan 2017; IPCC et al. 2021; Ruwanza et al. 2022), little to no information is available on exactly how these changes would impact livestock production (Thornton et al. 2014). Most studies on the subject focus on how season influences production in year- round mating systems (Adeleye 1984; Balogun et al. 1993; Babar et al. 2004; Mustafa et al. 2014; Đuričić et al. 2019), making the results essentially useless to commercial producers who have specific mating seasons.

Where specific weather variables are investigated, two challenges arise. Firstly, the specific variable is frequently predetermined prior to the investigation. It may therefore not be the most suitable variable for that particular situation (Van De Pol et al. 2016). The second, and more important problem, is that few of the available studies consider the long-term effects of weather on production performance. Most studies, especially those on heat stress, are primarily concerned with immediate impacts (Marai et al. 2007; Pluske et al. 2010; Al-Dawood 2017) or at best, effects occurring within the same reproductive cycle (Roth et al. 2004; Marai et al. 2007; Van Wettere et al. 2021). While this is undoubtedly a valid area of investigation, it ignores the potential impact so-called carry-over effects may have. Harrison et al. (2011) defined carry-over effects as processes that occur in one season that influence performance in the following season(s). Moore and Martin (2019) pointed out that these effects can affect fitness outcomes across an organism’s entire life cycle. Where carry-over effects have been studied in livestock, the focus has primarily been on nutritional, and not weather, effects (Broster et al. 1993; Chandra et al. 2011; Jørgensen et al. 2016).

This lack of knowledge regarding both immediate and potential longer term weather effects is hampering farmers’ decision-making as they are unable to accurately predict the influence of weather on current and future production potential. This study therefore aimed to explore the use of weather data as input variables in production prediction models to improve the decision-making of farmers. The objective was primarily to use the modelling process to quantify the degree of influence weather has on production performance at various times. The study consisted of two stages. In the first stage it was hypothesised that weather conditions during a given point in the production cycle could be used as predictors for performance at a later point in the same cycle. The hypothesis of the second stage was that weather effects could exert an influence on production in the following production cycle. The evaluation of these hypotheses is presented as the first steps in the development of potential predictive models for use by producers to ameliorate the impact of variable climate conditions.

## Methodology

The study made use of historical data collected between 1993 and 2021 on Elsenburg Research Farm (33° 50’ S, 18° 50’ E) in the Western Cape province of South Africa. Production data was sourced from a Merino flock kept on the farm while the weather data was provided by the Agricultural Research Council (ARC), which maintains an on-farm weather station. The Western Cape is a winter rainfall region (May-August) and is considered to have a Mediterranean climate (Zwane 2019). Average yearly rainfall during the study period was approximately 560 mm, meaning the region just escapes being considered semi-arid (Maliva and Missimer 2012).

The Merino flock from which the data was collected consists of two lines that have been divergently selected for reproduction (number of lambs weaned) from the same base population since 1986 (Cloete et al. 2017). There is an appreciable difference in reproduction between the two lines with the high line performing significantly better than the low line (Cloete et al. 2009, 2017; Naidoo 2012). According to Naidoo (2012) high line ewes birthed and weaned 1.33 and 1.05 lambs per ewe while the corresponding figures for low line ewes were 0.90 and 0.54. Production data from the flock is collected as part of standard flock management. The sheep were managed as one flock except during mating in January and February (summer) when the lines were separated into single-sire groups within selection lines. The flock was kept on irrigated kikuyu grass pastures in summer and a mixture of lucerne, kikuyu, medic and oats pastures in winter. The use of irrigated pastures is not a common practice in the Western Cape where the study was conducted, but the practice is employed in other parts of the country. If required, roughage bales were provided as supplementation during the dry period (summer and autumn). Strategic energy supplementation was also provided as necessary during extreme conditions.

Production traits included in the study were annual conception percentage, lambing percentage, multiple offspring percentage, survival percentage until weaning at ~ 100 days of age, average weaning weight and total weight weaned per ewe lambed. These were all considered as traits of the ewe. Conception percentage was calculated as number of ewes lambed divided by number of ewes mated while lambing and multiple offspring percentage were the number of lambs or multiples born divided by number of ewes lambed. Although the latter two traits are fairly similar, both were included here to determine their relative susceptibilities to weather effects and to evaluate how close the relationship between these traits was in this flock. Survival percentage was the number of lambs weaned divided by number of lambs born (both dead and alive). This trait, when considered as a trait of the ewe, is analogous to ewe rearing ability (Bunter et al. 2018). Defining it in this way also allows for the potential effect of weather conditions on pre- and post-natal ewe performance to be considered separately (lambing performance does not impact on weaning performance). Average weaning weight was the mean weaning weight of all lambs weaned while total weight weaned per ewe lambed was calculated as total weight of lamb weaned divided by number of ewes lambed. Again, defining the trait in this way allows the consideration of weather effects on weaning performance to be considered separately from potential effects on lambing performance. The weaning weights supplied in the dataset were pre-corrected for sex and age at weighing. In total 6779 individual ewe records, 4655 from the high line and 2124 from the low line, were condensed to provide yearly flock averages for each of the traits. Flock averages were used since individual ewe records are not available on most commercial farms. Stud breeders and participants in the National Small Stock Improvement Scheme may have these figures available, but they are only responsible for a limited proportion of total production (Schoeman et al. 2010; SA Stud Book 2015).

The weather data from the ARC were provided on a daily basis and included maximum and minimum temperature, maximum and minimum relative humidity, as well as rainfall. Wind speed and radiation were not available for the entire period and were therefore excluded. Daily data were edited and all days with missing observations for any of the weather variables were removed from the dataset. Temperature and relative humidity readings were then used to calculate temperature-humidity index (THI) values according to the formula used by Marai et al. (2001) where:

$$\begin{array}{l}THI=Temperature\:(^\circ\mathrm C)-\\\left[0.31-0.31\times Relative\:Humidity\:\left(\%\right)\right]\times\\\left[Temperature(^\circ\mathrm C)-14.4\right]\end{array}$$

Two THI values were calculated. The first used maximum temperature and minimum relative humidity values while the second was based on minimum temperature and maximum relative humidity. This was done since the data indicated an inverse relationship between temperature and humidity, where maximum temperature coincides with minimum relative humidity and vice versa. The two THI values therefore roughly corresponded to the highest daytime (*THId*) and lowest night-time (*THIn*) THI readings.

Thus, maximum (*Tmax*) and minimum temperature (*Tmin*), maximum (*RHmax*) and minimum relative humidity (*RHmin*), and daytime and night-time THI (*THId* and *THIn*) as well as rainfall (*Rain*) were the seven possible independent (predictor) variables on which the various production traits were regressed. The decision was made to use both minimum and maximum figures rather than an overall average as it provided a better indication of the environmental fluctuation animals were subjected to. It further provides for an indication of possible discomfort experienced by the animals at both the upper and lower levels of each weather variable. The mean value of each variable over the requisite period of time was calculated separately for each of the three sets of analyses. For mating period weather, mean values were calculated across January and February in each year, for lambing it was calculated across June and July, and annual averages were calculated for the final analysis.

Due to weather station malfunctions no data was available for the mating periods of 2000, 2001, 2015 and 2019 and these years were excluded from the regression of mating period weather variables on subsequent performance. Production and weather data for 25 years were therefore included in this dataset. In this analysis, the average weather conditions during a specific year’s mating period were linked with production output from that same year (production season) for statistical analysis.

The same procedure was followed to generate weather variables for the yearly lambing period. Lambing period weather data was only incomplete in 2000 and 2019, therefore this dataset contained data from 27 years. Conception rate was also excluded from the production traits for this analysis, as weather during lambing could not retro-actively affect this trait. These two sets of analyses addressed the first phase of the study, namely the influence of weather conditions on performance within a defined production cycle.

The second phase of the study focused on elucidating the effect of weather conditions across production cycles (carry-over effects) and to achieve this, production data of the current year was regressed on weather data from the preceding year, e.g. production figures from 2021 would be matched with weather data from 2020, thus allowing for a full year’s weather information to be considered for the prediction of production in the next season. No data was available for the year 2000 while 2001 and 2019 also had to be removed from the dataset due to an excessive number of missing observations. Twenty-five sets of observations for each line were constructed in this way. Due to the differences in reproductive performance (Cloete et al. 2009, 2017), the two lines were analysed separately in all procedures.

To overcome the problem of collinearity among the weather variables, a problem also highlighted by Van De Pol et al. (2016), a factor analysis was performed in Statistica 14 (TIBCO Statistica 2020) to determine the number of meaningful principal component factors. These components were identified by having an eigenvalue greater than one. The number of meaningful principal component factors were then used to estimate the optimal number of weather variables that would have to be included in standard regression analyses relating production traits as dependent variables to weather variables as independent variables. Exclusion criteria were set for these regression analyses to prevent correlated *y*-variables (*r* > 0.45) from being fitted in the same model. The other major advantage to following this method is that all available weather variables have an equal possibility of being found to influence production. In this way another of the major problems with these types of analyses highlighted by Van De Pol et al. (2016), namely variables being pre-selected prior to the analysis, is avoided. The model with the highest R^2^ value under these conditions was then identified while the root mean square error (RMSE) value was also included as an indication of model fit. Models were considered significant at a *P*-value of 0.05 or less.

## Results and discussion

The mean weather conditions experienced during each of the three time periods considered during the first phase of the study are presented in Table 1 below. These means clearly indicate the Mediterranean nature of the climate on Elsenburg Research Farm with moderate temperatures and winter rainfall being present.

**Table 1:** Mean weather conditions on Elsenburg research farm between 1993 and 2021 for each of the three time periods considered as potentially affecting Ewe performance in this study

Weather variables	Time period			
	Yearly	Mating (January-February)	Lambing (June-July)	
*Tmax* (°C)	23.76 ± 0.12	29.77 ± 0.19	17.93 ± 0.22	
*Tmin* (°C)	10.60 ± 0.10	14.68 ± 0.12	7.39 ± 0.16	
*Rain* (mm	578.53 ± 30.68	13.17 ± 2.17	200.56 ± 12.04	
*RHmax* (%)	92.18 ± 0.32	90.91 ± 0.35	86.38 ± 4.21	
*RHmin* (%)	46.57 ± 1.04	39.29 ± 0.83	50.91 ± 2.62	
*THId*	21.97 ± 0.09	26.76 ± 0.14	17.26 ± 0.18	
*THIn*	10.68 ± 0.10	14.66 ± 0.11	7.66 ± 0.19	

In the factor analysis for both mating and lambing period weather, three principal component factors displayed eigenvalues greater than one, indicating that they accounted for meaningful amounts of the variance in the data. Based on this number, a maximum of three weather variables had to be included in the regression procedure for each production trait. Due to the nature of weather conditions, it is inevitable that a certain measure of correlation exists among individual weather variables but, due to the exclusion criterion, the highest correlation between two variables included in a lambing period model in the high line was 34% and all variance inflation factor (VIF) values were below 1.27. A VIF value of less than 2.5 indicates that multicollinearity is not present in the model (Johnston et al. 2018).

The results from the regression analysis of mating period weather on production performance in the high line are summarised in Table 2.

**Table 2:** Regression equations describing various production traits in the high line of the Elsenburg Merino flock using weather conditions during mating as inputs

Production trait	Equation	*R* ^2^	RMSE	*P*-value
Conception percentage	$196.475-0.470RHmax-1.057THId-2.444THIn$	0.127	3.367	0.122
Lambing percentage	$\:247.349-1.291RHmax+5.948THId-9.556THIn$	0.332	7.581	0.009
Multiples percentage	$\:148.960-1.236RHmax+5.250THId-8.834THIn$	0.316	7.182	0.012
Survival percentage	$\:\:-33.108+3.300Tmax+0.214Rain+0.284RHmin$	0.068	6.304	0.223
Weight weaned/ewe lambed (kg)	$\:-16.654+0.073Rain+2.593THId-1.938THIn$	0.130	2.933	0.118

Models were developed for all production traits except average weaning weight but only the models for lambing and multiple offspring percentage, explaining 33.2% and 31.6% of the variation in reproduction respectively, were demonstrated to be significant (*P* < 0.05). The RMSE values also fell within acceptable limits.

With around a third of the variation in these two traits being attributable to weather conditions it proves that weather plays a significant role in affecting reproduction in the high line. The same weather variables, namely *RHmax*,* THId* and *THIn*, were included in the models for lambing and multiple offspring percentage. This is to be expected as an almost perfect correlation (*r* = 0.995) exists between these traits. The coefficients in these two equations are also fairly similar, indicating that both traits are affected to a similar extent by changes in weather as far as absolute performance is concerned. This does however mean that, relative to the mean performance level, multiple offspring percentage will be more susceptible to weather effects than lambing percentage. This is likely a result of the greater physiological demands placed on ewes that carry and rear multiple lambs, as the increased demand for bodily resources placed on these ewes could reasonably be expected to leave them more susceptible to environmental fluctuations.

Although caution should be exercised when attempting to interpret the relationship between single weather variables and production traits, an inverse relationship between reproduction and maximum relative humidity and night-time THI is present, while day-time THI is positively correlated with reproduction. This seems to suggest that ewes could tolerate, and might even prefer, increased thermal indices for optimal reproduction during a summer mating season. This is contrary to published findings (Van Wettere et al. 2021), but significantly more research would have to be done before a conclusive recommendation could be made. The negative coefficient of *THIn* indicates that ewes would reproduce better in a cooler environment. Of course, this is subject to the supposition that temperatures do not fall low enough to induce cold stress in the ewes. It could be hypothesised that, since heat stress is likely to occur during the hottest part of the day, the animals recover from short term heat stress during the night when it is cooler and more humid. Animals experiencing heat stress during the day will also increase their nocturnal activity (Al-Dawood 2017). If temperature and humidity increase during this period of either recovery or increased activity at night, it could potentially affect their ability to fully recover, thus impacting on their reproductive capacity. Again, further research would have to be done to confirm whether this is correct.

The negative coefficient for maximum relative humidity is contrary to the findings of Wilson and Sayers (1987), who reported a positive relationship between maximum relative humidity and litter size in West African Sahel type sheep that were farmed extensively under semi-arid tropical conditions in Mali. The difference may be ascribed to the previous study making use of data from a continuous mating system under extensive conditions where animals are free to mate throughout the year. This would presumably predispose the ewes to enter oestrus when grazing is plentiful, i.e. during or after the rainy season when humidity would be higher. Breed differences might also contribute to the contrast, with the longer wool of the Merinos potentially making them more likely to experience discomfort than the Sahel-type sheep with their hair coats.

It was found that conception percentage was largely independent of weather conditions during mating. Since both sperm production and follicle development take place prior to the mating period commencing (Evans 2003), it might be more informative to relate conception rate to weather conditions in the months preceding mating as there is evidence to suggest that conditions prior to mating may affect reproductive capacity in both rams (Gimenez and Rodning 2007; Marai et al. 2008; Shahat et al. 2020; Van Wettere et al. 2021; Moula et al. 2024) and ewes (Naqvi et al. 2004; Marai et al. 2008; Dobson et al. 2012; Romo-Barron et al. 2019; Van Wettere et al. 2021). No significant models could be developed for any of the weaning performance traits either. This is likely due to these traits indicating not just the performance of the ewes but also that of the lambs. Since the lambs were only born five months after mating it is extremely unlikely that weather conditions during mating would significantly affect their growth and survival.

The regression equations developed for the mating period in the low line are presented in Table 3. In this case models were developed for all traits, but only the lambing percentage model was deemed significant (*P* = 0.05) although multiple offspring percentage approached the threshold for significance (*P* = 0.055). The highest correlation between variables in the same model was 34.8% and all VIF values were below 1.22. Once again, the RMSE values were acceptable.

**Table 3:** Regression equations describing various production traits in the low line of the Elsenburg Merino flock using weather conditions during mating as inputs

Production trait	Equation	*R* ^2^	RMSE	*P*-value
Conception percentage	$260.173-0.428Rain-2.112RHmax+0.344RHmin$	0.154	9.070	0.091
Lambing percentage	$\:153.571+2.662Tmax-0.345Rain-6.548THIn$	0.205	8.498	0.050
Multiples percentage	$\:57.196+2.229Tmax-0.360Rain-5.934THIn$	0.197	8.223	0.055
Survival percentage	$\:-41.411+3.134Tmax+0.157Rain+0.394RHmin$	0.036	6.865	0.301
Average weaning weight (kg)	$\:18.572+0.058Rain-0.257RHmax+0.820THId$	0.020	2.332	0.346
Weight weaned/ewe lambed (kg)	$\:34.711+0.090Rain-0.112RHmax-0.227THId$	0.035	2.127	0.304

Unlike in the high line, *Tmax*, *Rain* and *THIn* were the three best predictors for lambing percentage. This equation only explained 20.5% of the variation in lambing percentage, more than 10% less than was explained in the high line. Although two variables differed between this and the high line equation, again it appears that ewes can tolerate more heat during the hottest part of the day as long as nights remain cool enough to allow them to recuperate from the thermal stress. The reason for the inverse relationship between rainfall and lambing percentage is unclear, although it could be speculated that rainfall during the mating period might affect mating behaviour.

Since the low line is genetically inferior as far as reproduction is concerned it was expected that weather conditions would have a greater effect on reproduction in this line, but the opposite was found to be true. It could be posited that this may be a result of the high line performing near or at their maximum genetic potential, meaning that any variation in performance is more likely to be due to environmental factors. Since these animals are high performing, they would require more favourable environmental conditions and be more susceptible to environmental fluctuations.

Contrastingly, the low line is at a lower performance level and this may actually help to buffer them against environmental fluctuations. The lower reproduction level is most likely accompanied by lower nutritional requirements and lower metabolic rates (reduced ovulation rate, lower pregnancy rates), meaning that the ewes are less reliant on an ideal environment to maintain an average production level.

Since weaning performance could not be satisfactorily modelled using mating period weather variables, lambing period weather variables were used as predictors instead. An attempt to explain variation in lambing performance based on weather during the lambing period was also made, but this was unsuccessful. Weather conditions during pregnancy were not considered in this study, but there is evidence to suggest that weaning performance may be affected by conditions during this period (Greenwood et al. 2009; Dado-Senn et al. 2020; van Wettere et al. 2021). Future studies expanding on the current one would be well advised to focus on the impact of environmental conditions during pregnancy on these traits.

Table 4 summarises the results of the regression analyses of ewe performance on lambing period weather conditions in the high line. Survival percentage could once again not be modelled, indicating that ewe rearing ability is entirely uncorrelated to lambing period weather conditions in the high line. The highest correlation between parameters for both the high and low line regressions was 36.9% and VIF values were below 1.17 in all cases.

**Table 4:** Regression equations describing weaning performance traits in the high line of the Elsenburg Merino flock using weather conditions during lambing as inputs

Production trait	Equation	*R* ^2^	RMSE	*P*-value
Average weaning weight (kg)	$\:36.551+0.009Rain-0.573THId-0.919THIn$	0.230	1.500	0.029
Weight weaned/ewe lambed (kg)	$37.906-0.095Tmax+0.006Rain-1.562THIn$	0.075	3.069	0.194

A significant model explaining 23% of the variation in average weaning weight was developed but weight weaned per ewe could not be satisfactorily modelled in the high line using lambing period weather variables as inputs. Rainfall was positively related to weaning weight. This potentially reflects the role that rainfall plays in fodder growth, which would affect the lambs’ growth by improving available fodder resources directly, while likely also stimulating ewe milk yield.

In the model, *THId* and *THIn* are inversely related to weaning weight. This indicates that an increase in THI levels during the lambing period is likely to lead to a reduction in weaning weight. Since lambs are born during winter this is not due to heat stress as temperatures are too low to cause heat stress. It does however indicate that lambs are unlikely to suffer from cold stress since the presence of cold stress would have led to a positive relationship between THI and production being present. However, this phenomenon must be investigated further to understand the relationship fully.

The regression equations for the low line are given in Table 5. All weaning performance traits were successfully modelled although only the model for survival percentage was significant (*P* = 0.048).

**Table 5:** Regression equations describing weaning performance traits in the low line of the Elsenburg Merino flock using weather conditions during lambing as inputs

Production trait	Equation	*R* ^2^	RMSE	*P*-value
Survival percentage	$88.322-0.041Rain+0.477THId-2.421THIn$	0.193	6.460	0.048
Average weaning weight (kg)	$\:33.480+0.009Rain-0.708THId-0.469THIn$	0.043	2.081	0.271
Weight weaned/ewe lambed(kg)	$29.140-1.069Tmin+0.063RHmin-0.377THId$	0.163	2.224	0.070

The model fitted to survival percentage explained 19.3% of the variation in the data through the fitting of *Rain*,* THId* and *THIn*, indicating that ewe rearing ability in the low line was affected by lambing period weather. Rainfall and night-time THI had an inverse relationship with survival percentage while day-time THI was directly related to the trait. Survival percentage is the inverse of lamb mortality, thereby explaining the relationship between the weather variables and the trait. An increase in rainfall, which is accompanied by a drop in temperatures and increased wind, will lead to increased incidences of cold stress in lambs (Nel et al. 2023). Less rain is therefore likely to promote lamb survival. An increase in maximum temperature and thus *THId* will reduce hypothermia and also improve survival. It is however unclear why a lower night-time THI will improve survival until weaning, although it could be theorised that the lower temperatures will drive ewes to seek shelter, thereby promoting lamb survival. Regardless, the effect of selection line can clearly be seen here as the rearing ability of the low line ewes is significantly impacted by weather conditions during lambing, whereas rearing ability in the high line ewes showed no significant weather effects.

This phase of the study assessed the utility of weather variables as predictors for flock performance within a production season. Although it seems feasible to do so, there is still a lot of development needed to render these models suitable for practical use. Firstly, this study was performed on a limited dataset from a single location with a moderate, Mediterranean climate. If it is at all possible, more generalized models must be developed to ensure a broader range of applicability across climatic zones. In an apparent contradiction, the envisaged models will have to consider genetic differences while also being climate or environment specific. Thus, models that are representative of the performance of a certain genotype under a range of conditions need to be developed.

Secondly, the predictive accuracy of the models will have to be improved to at least 70%. Although the models in this study provide valuable information, they do not consider any management or animal factors. Including management practices and animal factors such as age or genetic merit should improve the predictive accuracy of models for this purpose.

Finally, the relationships between the respective weather variables in the models will have to be explored and explained more fully. Clear-cut explanations for the relationship between weather variables and production traits are lacking in many cases. A more robust understanding of these relationships will improve the models and could potentially lead to the development of more comprehensive prediction models.

What these results do offer is a quantification of the relationship between weather conditions and flock reproductive performance in Merino ewes, something that was previously lacking. The presence of relationships between weather and reproduction within the same production season was clearly demonstrated, although the descriptive models lacked consistency across selection lines. It also appears that lambing performance is affected by weather conditions to a greater extent than weaning performance.

The results from the second phase of the study, focusing on the effect of weather conditions during the previous production cycle on performance in the current cycle are summarised in Table 6 (high line) and 7 (low line). The highest correlation coefficient between any two weather variables in the same model was 37.4% (*Rain* and *RHmin*) and the highest VIF in any model was 1.163. This once again indicates no collinearity being present.

**Table 6:** Summary of the linear regression models developed to describe flock production in the high line of the Elsenburg Merino flock using weather variables as independent regression variables

Production trait	Model equation	*R*^2^	RMSE	*P*-value
Conception percentage	$178.818-0.874RHmax-0.185RHmin$	0.060	3.584	0.195
Lambing percentage	$\:116.508+8.018Tmax-1.695RHmax$	0.217	8.756	0.026
Multiple offspring percentage	$\:-12.937+7.865Tmax-1.352RHmax$	0.218	8.264	0.026
Survival percentage	$\:49.894+3.138Tmax-0.481RHmax$	0.010	6.420	0.345
Average weaning weight (kg)	$\:20.518+0.005Rain-0.044RHmin$	0.180	1.337	0.043
Weight weaned/ewe lambed (kg)	$96.364-0.673RHmax-0.178RHmin$	0.082	2.732	0.150

Factor analysis indicated that only two weather variables were to be included in these regression procedures, as opposed to the three variables in the first phase of the study. All production traits in the high line could be successfully modelled using a bivariate model, although only the models for lambing percentage, multiple percentage and average weaning weight were significant (*P* < 0.05). The RMSE values included here indicated that all models fit the data.

Reproduction rate was significantly affected by maximum temperature and relative humidity. It was expected that both lambing percentage and multiple offspring percentage would be affected by the same weather variables as the two traits were 99.5% correlated. It was however interesting to note that, according to the magnitude of the coefficients in the equation, changes in the weather variables would affect multiple offspring percentage much more than lambing percentage, relative to average performance. The reasons for this have been discussed previously. Temperature was directly related to reproduction while relative humidity had an inverse relationship with the traits. This indicates that previous heat stress likely did not play a role in affecting current reproductive output in the high line of the Elsenburg Merino flock as the models indicate that the ewes would perform better under higher temperatures. With maximum and minimum temperature being moderately inversely correlated (*r*= −0.55), it is also possible that the increase in maximum temperature and potentially heat stress occurrence would be offset by a decrease in minimum temperature. However, this theory must be appraised carefully since the data was generated on an annual scale and the inverse correlation is therefore more likely to indicate an increase in extremes (higher maximum and lower minimum temperatures) over an entire year, rather than a shorter term (daily or weekly) relationship between the temperature readings.

An alternative explanation is that, since the weather data spans an entire season, the cumulative effect of cold during the winter months exceeded the influence of heat during the summer months. Since the ewes were mated in summer (January-February) and follicular development from the resting to the pre-ovulatory stages takes about six months (Cahill and Mauleon 1980; Evans 2003), it could be argued that follicular deposition and development, which plays an important role in reproduction rate, mostly took place during winter. This would then mean that warmer winters, which would require less energy expenditure by the ewes to maintain homeostasis, could potentially benefit reproduction rate. Warmer winters should also benefit pasture growth in the time leading up to mating in the new season, resulting in better fodder availability. The opposite of this (heat stress in summer adversely affecting ovulation in autumn) has been recorded in dairy cattle by Roth et al. (2004), proving the existence of temperature-related carry-over effects in reproduction. However, if this was the case it would be expected that the same weather conditions would also influence conception percentage, which is not reflected in Table 5. This phenomenon therefore warrants further study, potentially over a shorter timescale. From these results it does appear as if the high line is heat tolerant and would likely be able to maintain their level of reproduction even if mean maximum temperatures increase. Naturally, there will still be a threshold temperature beyond which they are not able to adapt, but that value would have to be determined through further study.

It is currently unclear why maximum relative humidity also affected reproduction, but it supports the findings of Wilson and Sayers (1987), who previously found that maximum relative humidity between 150 and 200 days prior to parturition significantly influenced litter size in West African Sahel type sheep in Mali. *RHmax* is also not correlated to any other variables which could potentially explain its effect on reproduction.

A significant model accounting for 18% of the variation in average weaning weight was also developed where *Rain* and *RHmin* were used as predictors. An increase in rainfall would lead to a slight increase in weaning weight while minimum relative humidity was inversely related to weaning weight. The effect of rainfall on weaning weight is likely attributable to the improvement in fodder availability and quality that is associated with increased rainfall (Dudney et al. 2017; Li et al. 2012). Not only would it benefit lambs directly by increasing the grazing available to them pre-weaning, it would also improve ewe performance throughout the year. Increased fodder availability should increase the nutritional plane of the ewe, allowing for a greater build-up of energy reserves during the dry period. This would then result in a positive carry-over effect between seasons (Harrison et al. 2011), improving the ewe’s ability to meet the energy demands associated with gestation and lactation in the season following the higher rainfall. Evidence supporting this hypothesis in dairy cattle is presented in the review of Jørgensen et al. (2016) where it was reported that improved nutrition pre-partum resulted in increased milk production and quality. Rainfall is also inversely correlated to maximum temperature and daytime THI and positively correlated with minimum temperature and night-time THI. Hence, an increase in rainfall would be accompanied by a more moderate thermal environment which could also be beneficial.

It is unclear why average weaning weight would benefit from a reduction in minimum relative humidity in the previous year. Since *RHmin* is inversely correlated with *Tmax* and directly correlated with *Tmin* and *THIn*, it is possible that a reduction in *RHmin* indicated a slightly warmer environment, rather than any direct influence on the trait, but this supposition will have to be investigated further.

As indicated in Table 7 below, all traits except survival percentage could be modelled in the low line, but only the models for conception percentage (*P* = 0.02) and average weaning weight were significant (*P* = 0.007). As before, the RMSE values indicated acceptable model fit.

**Table 7:** Summary of the linear regression models developed to describe flock production in the low line of the Elsenburg Merino flock using weather variables as independent regression variables

Production trait	Model equation	*R* ^2^	RMSE	*P*-value
Conception percentage	$364.278-0.026Rain-2.966RHmax$	0.237	8.928	0.020
Lambing percentage	$161.001-0.022Rain-0.346RHmin$	0.140	8.338	0.073
Multiple offspring percentage	$58.453-0.022Rain-0.298RHmin$	0.149	7.940	0.065
Average weaning weight (kg)	$\:16.061+0.009Rain-0.032RHmin$	0.306	1.734	0.007
Weight weaned/ewe lambed (kg)	$8.912-0.066RHmax+1.453THIn$	0.008	2.325	0.350

Conception percentage was dependant on rainfall and maximum relative humidity in the preceding year, with 23.7% of the variation in conception rate being accounted for by fitting the model. Both weather variables were inversely correlated with conception. It would be expected that rainfall, through its influence on fodder availability (Dudney et al. 2017; Li et al. 2012), would be positively related to conception but this was not the case. Although the models for lambing and multiple offspring percentages in Table 7 were non-significant (*P* > 0.05), an inverse relationship between rainfall and these traits were also indicated by the models. With *Rain* also being correlated to a more moderate thermal environment (data not shown), it is unclear why increased rainfall would impair conception. Since *RHmax* is also inversely correlated to conception it appears that low line ewes preferred a drier, less humid environment for optimal reproduction, but currently no plausible explanation for this preference can be provided.

As in the high line, average weaning weight was modelled using *Rain* and *RHmin* as predictors. Almost a third (30.6%) of the variation in weaning weight could be explained by this combination. Weaning performance in the low line thus appears to be more susceptible to fluctuations in weather conditions in the previous year than in the high line. It is possible that this is a result of the ongoing selection in the flock with high line ewes being selected for number of lambs weaned (Cloete et al. 2017). Lambs from the high line are therefore likely to be genetically superior for weaning performance and potentially less susceptible to environmental effects. How much of the difference in performance or susceptibility is a result of carry-over effects in the ewes and how much is due to direct lamb performance is beyond the scope of this study to determine although it certainly offers opportunities for further research.

Overall, weather patterns in one production season significantly influenced some of the production traits in the subsequent production season. The degree of influence varied between traits and lines but is sufficiently large (18–30%) to warrant further study. This also confirms the presence of weather-related carry-over effects in this flock and can serve as the starting point for further investigation into the topic. It has to be emphasised that this research was not concerned with examining the mechanism by which weather conditions in a previous season affected production performance in the current season, but only in identifying the possible existence and magnitude of such effects.

Now that the relationship between weather and production has been somewhat illuminated, it could be speculated as to whether weather effects act directly on the animal to affect performance or whether an indirect action is present. Direct effects would likely take the form of heat (Pérez et al. 2020; Van Wettere et al. 2021; Pryce et al. 2022) or cold stress (Abdelqader et al. 2017; Piirsalu et al. 2020; Nel et al. 2023) affecting reproduction (Silanikove 2000; Roth et al. 2004; Dado-Senn et al. 2020) or survival (Abdelqader et al. 2017; Nel et al. 2021). Indirect effects would occur as a result of weather conditions affecting the availability of environmental resources e.g. fodder (Li et al. 2012; Dudney et al. 2017). There is evidence present in literature supporting both the hypothesis for direct (Roth et al. 2004; Dado-Senn et al. 2020) and indirect (through nutrition) carry-over effects (Broster et al. 1993; Chandra et al. 2011; Parr et al. 2015; Jørgensen et al. 2016) in livestock. These hypotheses are also applicable to the mechanism of influence that weather conditions exert on production within the same production season as seen in phase one of this study. It seems most likely that a combination of direct and indirect effects is responsible for the weather impacts recorded in this study, but further studies are advised.

It can be concluded that the influence of weather variables on traits of economic importance is large enough to be of concern to producers and that changing weather patterns can significantly alter farm production and profitability, both in the short and longer term. This premise can serve as the basis for further studies, potentially linking predicted changes in weather and climate to expected changes in sheep production. Before this can be done, more complete predictive models that consider various animal and management factors on production along with the weather influences will first have to be developed.

## Conclusion

The models constructed in this study illustrate the basic relationship between various weather variables and production traits in a Merino flock both within and between production seasons. The influence of weather on some production traits can be considered significant and, even if the same management regime is followed annually, producers can still expect significant variation in their production figures stemming from weather conditions. These effects seem to be more concentrated around lambing performance (lambing and multiple offspring percentages) than weaning performance (survival until weaning, average weaning weight). It also highlights that these effects will not be confined to more marginal, arid production zones but will also affect production in the more moderate climatic zones of South Africa.

The successful fitting of these models indicated that the basic premise of using weather variables to predict production output is sound and that further exploration in the field is warranted. Further work needs to be done to refine the models and relationships between the various weather variables while datasets collected over longer time periods, in different areas and across multiple breeds should also be analysed to confirm the direction and magnitude of the correlations between weather and production reported here.

## Data Availability

The datasets analysed during the current study are available from the corresponding author on reasonable request and subject to institutional approval.
